# Medical Text Classification Using Hybrid Deep Learning Models with Multihead Attention

**DOI:** 10.1155/2021/9425655

**Published:** 2021-09-23

**Authors:** Sunil Kumar Prabhakar, Dong-Ok Won

**Affiliations:** ^1^Department of Artificial Intelligence, Korea University, Seongbuk-gu, Seoul 02841, Republic of Korea; ^2^Department of Artificial Intelligence Convergence, Hallym University, Chuncheon, Gangwon 24252, Republic of Korea

## Abstract

To unlock information present in clinical description, automatic medical text classification is highly useful in the arena of natural language processing (NLP). For medical text classification tasks, machine learning techniques seem to be quite effective; however, it requires extensive effort from human side, so that the labeled training data can be created. For clinical and translational research, a huge quantity of detailed patient information, such as disease status, lab tests, medication history, side effects, and treatment outcomes, has been collected in an electronic format, and it serves as a valuable data source for further analysis. Therefore, a huge quantity of detailed patient information is present in the medical text, and it is quite a huge challenge to process it efficiently. In this work, a medical text classification paradigm, using two novel deep learning architectures, is proposed to mitigate the human efforts. The first approach is that a quad channel hybrid long short-term memory (QC-LSTM) deep learning model is implemented utilizing four channels, and the second approach is that a hybrid bidirectional gated recurrent unit (BiGRU) deep learning model with multihead attention is developed and implemented successfully. The proposed methodology is validated on two medical text datasets, and a comprehensive analysis is conducted. The best results in terms of classification accuracy of 96.72% is obtained with the proposed QC-LSTM deep learning model, and a classification accuracy of 95.76% is obtained with the proposed hybrid BiGRU deep learning model.

## 1. Introduction

There is a huge increase in the total number of electronic documents available online due to the development of information and Internet technology. This huge and unstructured form of text enables the automated text classification to a great extent [1]. In the field of NLP, text classification is one of the most important fields, and it helps in the assignment of the text documents to proper classes depending on their content. Many challenges and solutions are exhibited by the publicly available documents, and its classification is mainly intended for web classification, unstructured text classification, sentiment classification, spam e-mail filtering, and author identification [2]. Supervised classification techniques, like support vector machine (SVM) or naïve Bayesian classifier (NBC), are employed for extraction of features when done by the most common bag of words approach [3]. As some words can be neglected easily along with the small training data here, it may suffer from sparsity problem. Therefore, recent studies concentrate on focusing of more complex features. In the text classification field, a special emphasis is always given to the medical text classification as a lot of medical records along with medical literature are contained in the medical text [4]. The medical records include the doctor's examination, diagnosis procedures, treatment protocols, and notification of improvement of the disease in the patient. The entire medical history along with the prescription effect of the medicine on the patient is also stored in the medical record. The medical literature includes the oldest and recent documents of the medical techniques used for diagnosis and treatment of a particular disease [5]. Both these two information resources are very important in the field of clinical medicine. Due to the advent of information technology, tremendous quantity of electronic medical records and literature have been found online, which provides good resources of data mining in the medical field. Text classification in medical field is quite challenging because of two main issues: first, it has a few grammatical mistakes, and second, a lot of medical techniques are presented in the text [6]. With the advent of deep learning, such as convolutional neural networks (CNN) and recurrent neural network (RNN) being used widely in image, signals, and other applications, it has been equally successful in medical text classification [7].

Medical data can be classified on word, sentence, and even document levels in some works [8]. A good amount of medical data is available online, and these data provide useful information about the disease, symptoms, treatment, patient history, medication, and so on. To imbibe the most useful information, they need to be classified into their respective classes. An important step towards further implementation, such as classification and design of an automated medical diagnosis tool, is enabled by this task. Only very few works with respect to medical text classification has been proposed in literature, and only a handful amount of works have addressed multiclass medical text classification and some works have concentrated on binary medical text classification [9]. The majority of the medical text classification models are either on the word level or the sentence level classification rather than the document level classification. A recent comprehensive survey on text classification from shallow to deep learning was discussed in [8], and a survey on text classification algorithms was thoroughly analyzed recently in [9]. These two survey papers are very much useful as all the past techniques, associated working methodologies, datasets analysis, and comparison of all the results along with the possible future works are discussed well, thereby making it a nonnecessity for other authors to repeat the past works over and over again. However, a few essential works, which deal with medical text classification, are discussed in this work as follows. A famous work for medical text classification, which is being cited by almost every researcher in the medical text classification, was done by Hughes et al., where they have used more complex schemes to specify the classification features using CNN [10]. A systematic literature review along with the open issues present in it, exclusively in the field of clinical text classification research trends, was analyzed comprehensively by Mujtaba et al. [11]. A novel neural network-based technique using a BiGRU model [12], a paradigm using weak supervision and deep representation [13], a rule-based feature representation with knowledge guided CNN [14], a deep learning-based model using hybrid BiLSTM [15], and an improved distributed document representation with medical concept description for traditional Chinese medicine clinical text classification [16] are some of the famous works in the medical text classification. A cancer hallmark text classification using CNN was proposed by Baker et al., where the medical datasets were thoroughly investigated [17]. Other works in medical text classification, providing some interesting results, include the integration technique of attentive rule construction with neural networks [18], genetic programming with the data driven regular expressions evolution methodology [19], improving multilabel medical text classification by means of efficient feature selection analysis [20], multilabel learning from medical plain text with convolutional residual models [21], and ontology based two-stage approach with particle swarm optimization (PSO) [22]. A medical social media text classification integrating consumer health technology [23], NLP-based instrument for medical text classification [24], efficient text augmentation techniques for clinical case classification [25], and hybridizing the idea of deep learning with token selection for the sake of patient phenotyping [26] are some of the applications related to medical text classification in general health technology aspects. The application of medical text classification in clinical assessments deals with the works, such as time series modelling using deep learning in the intensive care unit (ICU) data [27], phenotype prediction for multivariate time series clinical assessment using LSTM [28], hybridizing of RNN, LSTM, GRU, and BiLSTM for extraction of the clinical concept from texts [29], and identifying of the depression status in youth using unstructured text notes along with deep learning in [30]. An automatic text classification scheme, known as FasTag, which deals with unstructured medical semantics, was proposed recently in [31]. Similarly, many NLP tools are available in literature with specific observations, source codes, frameworks, and licenses, such as CLAMP, MPLUS, KMCI, SPIN, and NOBLE [32]. Every work proposed in literature has its own merits and demerits. No method is consistently successful at all times and no method is a consistent failure too. On analyzing this important point, after several trial-and-error attempts, in this work, two efficient deep learning models for medical text classification are proposed as a boost to the existing methods reporting some good results. Therefore, the main contributions in the paper are as follows:

- A quad channel hybrid LSTM deep learning model has been implemented, and to the best of our knowledge, no one has ever developed, such a type of model for medical text classification. The main intention to develop a quad channel hybrid LSTM model is because, with four input channels, the characteristic diversity of the input can be greatly improved, thereby enhancing the classification accuracy of the model.
- A hybrid BiGRU model with a multihead attention is also successfully developed, and the primary intention to develop such a model is that the effective features in multiple subspaces can be well explored and the concatenation of the convolutional layers with the BiGRU layer can definitely provide good classification accuracy.

The organization of the paper is as follows. In Section 2, the two deep learning models for medical text classification are proposed, and the results and discussion are present in Section 3, followed by the conclusion in Section 4.

## 2. Design of Deep Learning Models for Classification of Medical Text

The hybrid deep learning models developed for medical text classification include two methods such as a quad channel hybrid LSTM model as a first method and hybrid BiGRU model as the second method.

### 2.1. Proposed Method 1: Quad Channel Hybrid LSTM Model

Generally, there is a limitation of the semantic features as only the word level embedding is used often by the traditional CNN and RNN networks. There is a very limited capability when utilized by these models especially when the semantics has to be determined by these words. Therefore, expansion of channels is quite necessary, and the usage of multilevel embedding is required so that the characteristic diversity of the input is improved. For every word in the text, the relative importance is quite contrasting from the modality, which is conveyed. Few words can give a lot of contribution to modality, while some words have less contribution to modality. Therefore, to learn the characteristics of every word in a detailed manner, hybrid attention is added after LSTM, so that a tradeoff is achieved for various words with contrasting emotions. Thereby, the learning potential of the LSTM representation is improved. Overall, the unique characteristics in the learning of neural network representation are also improved. This specific aspect helps to refine the generalization, so that the overfitting can be easily prevented [33]. The division of the proposed model is done in the following parts, such as word embedding, hybridizing of CNN with LSTM, and hybrid attention scheme followed by the design of quad channel LSTM.

#### 2.1.1. Word Embedding

**Figure 1:**
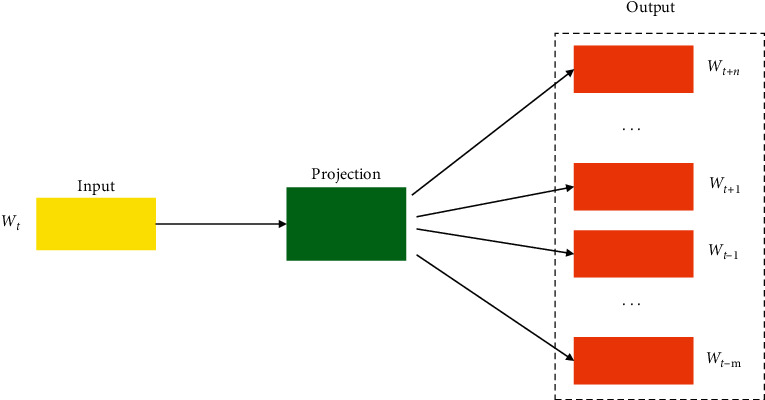
Structure of word embedding module.

For the representation of the word, an unsupervised learning algorithm called GloVe is utilized in order to obtain vector representations for words [34]. It is a count-based word representation natural language processing tool, and it utilizes the overall statistics. In between the words, the main semantic properties are captured by a vector of real numbers. By analyzing the Euclidean distance or the cosine similarity, the semantic similarity between the two words is computed easily. Word and character levels are two kinds of word segmentation considered in this model. Word2vec model proposed in [34] uses the related attributes between words, so that the semantic accuracy is increased. To deal with the dimensionality problem, a low dimensional space representation is utilized. CBOW and skip-gram are the two architectures used for word embedding in Word2vec. The surrounding words are utilized to predict the center word by CBOW method, and the central words are utilized to predict the surrounding words by skip-gram method. CBOW is fast in terms of swiftness for training the word embedding when compared to skip-gram. Skip-gram seems to be better with regard to accuracy when the semantic detail is expressed. Therefore, to train the word embedding, Word2vec model dependent on skip-gram is utilized in this paper.  Figure 1 expresses the structure of a word embedding module.

#### 2.1.2. CNN with LSTM Module

**Figure 2:**
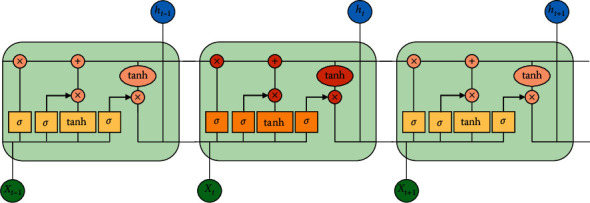
Illustration of a typical LSTM unit with suitable inputs and outputs.

One of the primary algorithms of deep learning techniques is CNN. It is a famous feed forward neural network with a deep structure, which has convolution calculation, and it has been successfully implemented in computer vision and NLP. In this work, the hybrid combination of CNN along with LSTM is utilized. For processing the sequential data, RNN is used widely. The past output and the current input are concatenated together by this RNN model. The activation function tanh is used to control it, so that the sequence states can be considered. At a time *t*, the RNN derivative will spread and communicate to time *t* − 1, *t* − 2, ..., *l*, thereby leading to the existence of a multiplication coefficient. Gradient explosion and disappearance occur when there is continuous multiplication occurring. During the forward process, the input of the start sequence has a very small or negligible effect on the late occurring sequences, and therefore, it is considered as a main problem of loss distance dependence. By means of introducing several gates, LSTM problem can be easily solved. The memorization of the input is done in a selective manner by the LSTM gate structures [35]. The memorization of the most vital information is done, and the less important information is forgotten completely. Thereby, the assessment of the next new information that could be saved in the current state is generated successfully. To a sigmoid function, the preceding state output *h*_*t*−1_ and the contemporary input in a function *X*_*t*_ are fed as an input so that a value between 0 and 1 is generated, thereby determining the current new information that could be retained easily. The complete state *C*_*t*_ of the next moment is obtained with the help of forget gate and the input gate, and it is utilized for the inception of the hidden layer *h*_*t*_ of the succeeding state, thereby forming the output of the present unit. The determination of the output is done by the output gate with respect to the information obtained from the cell state. A sigmoid function homogeneous to input gate, which generates a value *o*_*t*_ between 0 and 1, shows the amount of cell state information determined to project it as output. When the multiplication of the cell state information happens with *o*_*t*_, it is activated by means of utilizing tanh layer, and so, the output details of the LSTM representation *h*_*t*_ are modeled.  Figure 2 shows the illustration of a typical LSTM unit with suitable inputs and outputs. For the LSTM, the corresponding alliances between the various gates are mathematically expressed as follows:

(1) $$\begin{array}{c} z_{t}=\tanh\left(W_{z}\left[h_{t-1},X_{t}\right]+b_{z}\right),\\ i_{t}=sigmoid\left(W_{i}\left[h_{t-1},X_{t}\right]+b_{i}\right),\\ f_{t}=sigmoid\left(W_{f}\left[h_{t-1},X_{t}\right]+b_{f}\right),\\ o_{t}=sigmoid\left(W_{o}\left[h_{t-1},X_{t}\right]+b_{o}\right),\\ c_{t}=f_{t}\cdot C_{t-1}+i_{t}\cdot z_{t},\\ h_{t}=o_{t}\cdot \;\tanh\left(c_{t}\right). \end{array}$$

**Figure 3:**
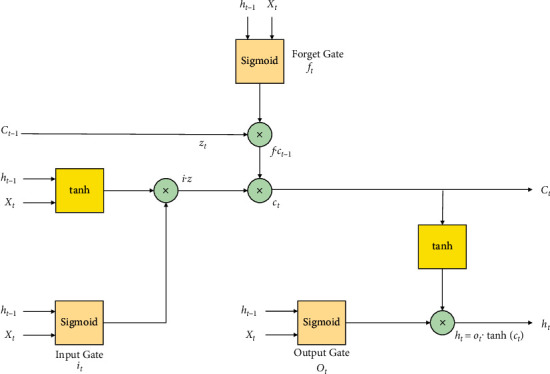
Illustration of a LSTM unit utilized in this work.

Figure 3 expresses the illustration of a LSTM unit utilized in this work. The gradient problem explosion will surely occur if the length of the input sequences is longer, thereby making it hectic to learn the information from a long-time context. To solve this issue, the most popular variation of RNN that can be utilized is LSTM, and by means of launching a gate structure in every LSTM unit, this problem can be easily solved. The discarding information from a cell state is decided by the forget gate, and the assessment of new inputs is determined by the input gate. Depending on the present state of the cell, the determination of output value is done based on the information added to the cell state. A four-channel mechanism is introduced in the CNN-LSTM model by means of giving multiple labels of embeddings as input simultaneously at a given instant of time, so that multiple aspects of features are acquired. Therefore, the extraction of both word level and character level features can be done easily and at the same time. Based on the embedding granularity, the structure is split into the character and word levels. In each channel, the structure of model is sequential, and it is divided into two unique but different parts, such as CNN and LSTM neural network. For the input sequence *X*, the convolution result *c* is computed along with the convolution kernel *K* and is mathematically represented as

(2) $$\begin{array}{c} c=conv\left(X,K\right)+b. \end{array}$$

For simplification of the representation, the LSTM procedure is unified as *LSTM*(*x*). Series and parallel structures can be utilized for CNN and LSTM neural networks. Generally, series structures are commonly used in spite of the information loss due to the nature of the convolution process. Many time series characteristics are lost with the series structure, and so, compressed information is received with LSTM neural network. Therefore, series structure is replaced by parallel structure, and the results obtained are pretty good. In every channel, the recording of the structure is done, and it is expressed as

(3) $$\begin{array}{c} \text{channel}\left(x\right)=\left[conv\left(x\right)⊕\text{LSTM}\left(x\right)\right]. \end{array}$$

The basic explanation of the character and word levels is obtained from (3). With *x* representing the input and the output, expressed as *C*_*out*_ and *W*_*out*_, it can be expressed as follows:

(4) $$\begin{array}{c} C_{out}={\text{channel}}_{\text{embedding}}=v_{w}\left(x\right),\\ W_{out}={\text{channel}}_{\text{embedding}}=v_{c}\left(x\right). \end{array}$$

The word level embedding vectors trained is expressed as *v*_*w*_, and the character level embedding vector trained is expressed as *v*_*c*_, respectively. The interpretation and outcome of the output of the four channels are merged as a hidden layer output and is represented as

(5) $$\begin{array}{c} h=\left[C_{out}⊕W_{out}\right]. \end{array}$$

To the fully connected (FC) layer, this hidden layer result is sent, and finally for the classification output, the Softmax layer is used and is represented as

(6) $$\begin{array}{c} \overset{⌢}{y}=\text{softmax}\left(\text{dense}\left(h\right)\right). \end{array}$$

The four-channel representation is explained in the following sections, respectively.

#### 2.1.3. Hybrid Attention Model

A vital constituent of the dynamic pliable weight structure is represented by the weight score *w* and its computation is expressed as

(7) $$\begin{array}{c} e_{i}=v_{a}^{T}\tanh\left(W_{r}h_{i}+b\right),\\ h_{i}=\langle h_{t}^{\prime }:c_{t}\rangle , \end{array}$$

where *h*_*t*_′ indicates the LSTM output at a specific time *t*, *h*_*i*_ expresses the hidden layer output, *c*_*t*_ indicates the states in LSTM, *v*_*a*_ indicates the random initialization vector, *b* represents the bias, which is randomly initialized, and *W*_*r*_ indicates the random initialization weight matrix. The computation of score *w* is done as follows:

(8) $$\begin{array}{c} w=\frac{\exp\left(e_{i}\right)}{\varepsilon }\left[\sum_{k=1}^{T_{x}}\exp\left(e_{i}k\right)\right], \end{array}$$

where the sequence length is expressed as *x*.

The dynamic adaptive weight is weighted to an output vector *c*_*i*_ and is represented as

(9) $$\begin{array}{c} c_{i}=\sum_{j=1}^{T_{x}}w\cdot h_{j}. \end{array}$$

#### 2.1.4. Design of the Quad Channel Hybrid LSTM Model

**Figure 4:**
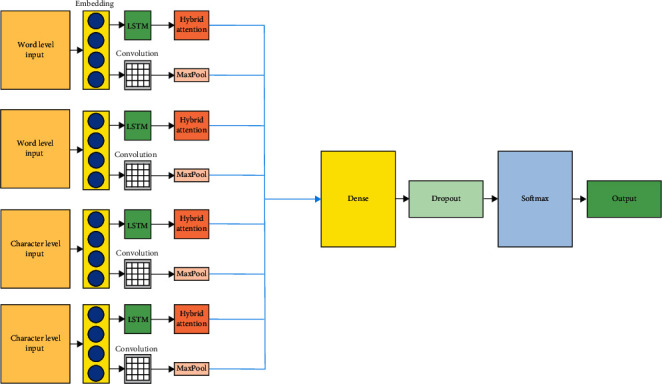
Proposed quad channel hybrid attention model.

The input text is first embedded, and then, the vector representation of these sequences is obtained to get a better semantic depiction and extricate the best text features. After the vector portrayal of these sequences are obtained, then these sequences are convolved by utilizing the convolution layer. The word-level semantic features can be well extracted by this model, and so, the input data along with the output size can be reduced by means of mitigating the overfitting aspect. The convolutional layer processes the data efficiently, and it sends it to the LSTM layer, so that the timing characteristics of the data can be well analyzed. Therefore, to increase the classification accuracy and avoid the secondary information of context semantics, this architecture is achieved.  Figure 4 illustrates the quad channel hybrid attention model.

### 2.2. Proposed Method 2: Hybrid BiGRU Model with a Multihead Attention

To the word embedding layer, the data processing results are fed as input, and the corresponding word vectors are obtained as output, which has rich semantics and a very low dimensionality. To extract the local features, CNN has a very strong ability, and parallel computing is enabled by it, so that a high training speed is achieved. To get the feature maps, multiple filters with suitable filter sizes are adopted. The features obtained from convolution are dealt with much efficiency here by means of applying maximum pooling and average pooling approaches, so that a good feature information is captured, and then, it is concatenated thereby the sentences are represented well. In order to get exact and more accurate semantic information, BiGRU is applied, so that the context information is extracted. The main reason for implementing the BiGRU is due to the inability of CNN to capture context information and the gradient explosion problem caused by the simple RNN. In multiple subspaces, more effective and potential features can be obtained by the multihead attention rather than using single-head attention. The multihead attention layer outputs are nothing but the weighted word vector representation. Many global features are obtained by means of implementing the maximum and average pooling techniques, so that the word vector can be represented more accurately. Depending on the distinct attributes of CNN, BiGRU along with the multihead attention, the features are merged or concatenated as final features, and then, it is fed to the FC layer. Finally, the Softmax classifier is utilized to perform the classification process.

#### 2.2.1. CNN and Text CNN

**Figure 5:**
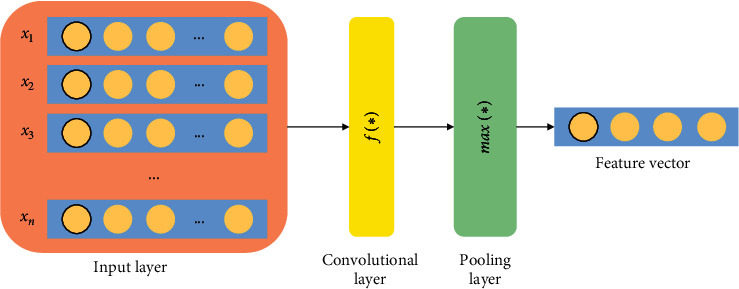
Structure of a CNN.

By means of imitating the biological visual perception mechanism, CNN was constructed, and so, both supervised learning and unsupervised learning are done easily [36]. With a very small amount of calculation, the lattice point features are obtained by CNN as the sparsity of the connections between the layers is enabled along with the sharing of parameters of convolutional kernel in the hidden layer.  Figure 5 explains the structure of the CNN and it comprises of input layer, convolutional layer, and pooling layer along with a FC layer.

A text classification model known as text CNN is developed in [37] by making some preliminary adjustments or modifications in the input layer of the traditional CNN, and this work has been partly inspired by it and has been used in our work too. After the padding, the length of the sentence is considered to be *n*, the filter size is denoted by *h*, and the word embedding dimension is denoted by *d*. The successful merging of words such as *x*_*i*_, *x*_*i*+1_, ..., *x*_*i*+*h*−1_ is expressed in every sentence as *x*_*i*:*i*+*h*−1_. By means of using a nonlinear function, the resulting of a feature *t*_*i*_ is obtained from a collection of words *x*_*i*:*i*+*h*−1_ and it is represented as follows:

(10) $$\begin{array}{c} t_{i}=f\left(wgx_{i+h-1}+b_{i}\right). \end{array}$$

**Figure 6:**
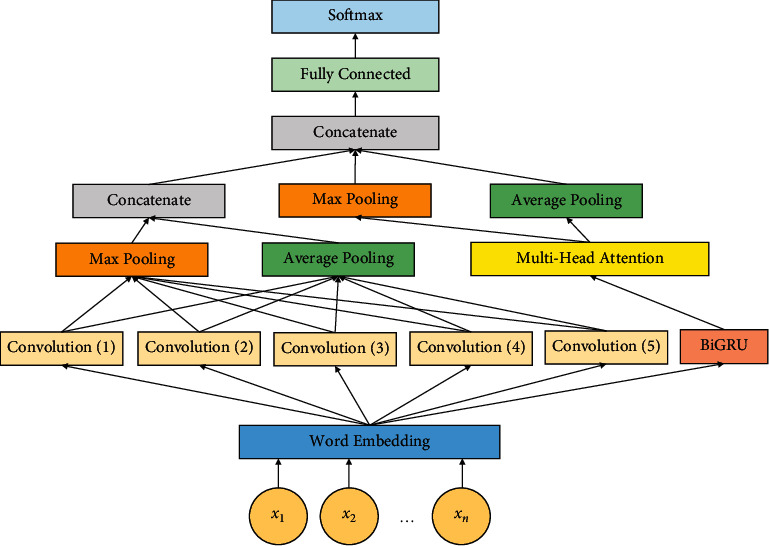
Proposed hybrid BiGRU deep learning model.

The bias term is represented as *b*_*i*_ and *w* ∈ *ℜ*^*hd*^ is a filter kernel. In the sentence representation [*x*_1:*h*,_*x*_2:*h*+1_, ..., *x*_*n*−*h*+1_]^*T*^, this filter is used to each window of words, so that a feature map [*t*_1_, *t*_2_, ..., *t*_*n*−*h*+1_]^*T*^ is obtained, and thus, the feature extraction from a filter is expressed by the previously mentioned process. The extraction of local features of various sizes is done by means of utilizing the diverse characteristics of the different filter kernel size. In this work, maximum and average pooling techniques are implanted to the features, which are obtained from the convolution layer, so that more features are extracted.  Figure 6 expresses the proposed hybrid BiGRU deep learning model architecture.

#### 2.2.2. Description of BiGRU Utilized in the Work

**Figure 7:**
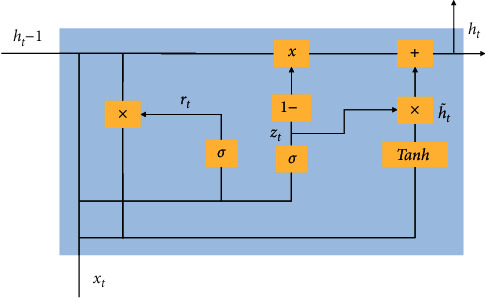
An illustration of a GRU.

A famous kind of RNN is GRU [38], and to fathom issues like long-term memory along with gradients in the backpropagation process, this technique was utilized to solve the problem and it is more or less similar to LSTM. With sequential data as input, recursion is performed in the evolutionary direction of sequences by this class of RNN and the connection of all the neurons are in a chain. The information can be well received by the neurons from their own historical moments because of the cyclic factors addition in the hidden layer. The traits of sharing both memory and parameters are present in the RNN. In order to deal with the nonlinear feature learning of several data, RNN seems to be quite superior. The RNN gradient disappearance is a huge problem, and so, long-term historical load features cannot be learnt and LSTM is proposed by researchers, as in between the long short-term sequence data, the correlation information can be easily learnt. To deal with LSTM and its huge parameters along with a very slow or moderate convergence rate, GRU has been procured. Thus, a famous alternative of LSTM is GRU as it has very less parameters and can achieve a high convergence rate along with a good learning performance too [38]. Internally, the GRU model comprises of update gate and reset gate. The input gate and forget gate of LSTM are replaced by the update gate of GRU. The effect of output information of the hidden layer neuron is represented by the update gate at the preceding moment in the hidden layer neurons of the present moment. The influence degree is pretty high when the value of updating gate is large. At the preceding moment, the hidden layer neuron outputs are indicated by the reset gate, and less information is generally ignored when the reset gate value is large. A typical illustration of a GRU is depicted in Figure 7.

Using the following formulae, the hidden layer unit can be computed:

(11) $$\begin{array}{c} z_{t}=\sigma \left(W_{z}.\left[h_{t-1},x_{t}\right]\right),\\ r_{t}=\sigma \left(W_{r}.\left[h_{t-1},x_{t}\right]\right),\\ {\tilde{h}}_{t}=\tanh\left(W.\left[r_{t}*h_{t-1},x_{t}\right]\right),\\ {\tilde{h}}_{t}=\left(1-z_{t}\right)*h_{t-1}+z_{t}*{\tilde{h}}_{t}. \end{array}$$

where *z*_*t*_ represents the update gate and *r*_*t*_ represents the reset gate.

**Figure 8:**
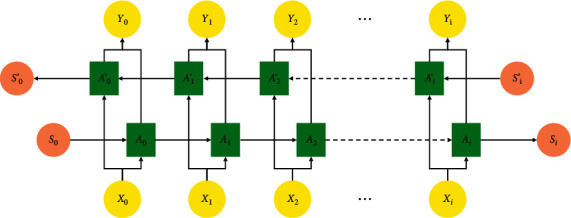
Illustration of a BiGRU.

The sigmoid function is represented by *σ*. The hyperbolic tangent is expressed by tanh. The training parameter metrics considered here are *W*_*r*_, *W*_*z*_ along with *U*_*r*_, *U*_*z*_, and *U*. The training parameter metrics *W* and *U*, resetting gate *r*_*t*_, input *x*_*t*_ at the current moment, and output *h*_*t*−1_ at the previous moment of the hidden layer neuron are used to assess the candidate activation state ${\tilde{h}}_{t}$ at the present moment. To grasp the association between current load along with the past and future load effecting components, a good capacity is present in the BiGRU network as the deep features of the load data can be conductively extracted. The structural representation of BiGRU is shown in Figure 8.

Its computations are as follows:

(12) $$\begin{array}{c} Y_{2}=g\left(VA_{2}+V^{\prime }A_{2}^{\prime }\right). \end{array}$$

The computation of *A*_2_′ is as follows:

(13) $$\begin{array}{c} A_{2}=f\left(WA_{1}+Ux_{2}\right),\\ A_{2}^{\prime }=f\left(W^{\prime }A_{3}^{\prime }+Ux_{2}\right). \end{array}$$

The hidden layer value *S*_*t*_ is highly affiliated to *S*_*t*−1_ in the forward calculation. The hidden layer value *S*_*t*_ is also highly concomitant to *S*_*t*−1_ in the reverse calculation. Depending on the success of both the forward and reverse calculations, the computation on final output is obtained. For the bidirectional RNN, the computation is as follows:

(14) $$\begin{array}{c} o_{t}=g\left(VS_{t}+V^{\prime }S_{t}^{\prime }\right),\\ S_{t}=f\left(Ux_{t}+WS_{t-1}\right),\\ S_{t}^{\prime }=f\left(U^{\prime }x_{t}+W^{\prime }S_{t-1}^{\prime }\right). \end{array}$$

#### 2.2.3. Implementation of Cross-Entropy Loss Function

For classification issues, the implementation of the cross-entropy loss function is usually done [39]. The probability of each category is computed by the cross-entropy, and it materializes with sigmoid or softmax function mostly. Sigmoid function is usually expressed as follows:

(15) $$\begin{array}{c} \sigma \left(z\right)=\frac{1}{1+e^{-z}}. \end{array}$$

The following function is obtained once the sigmoid function *σ*(*z*) is derived and represented as

(16) $$\begin{array}{c} \sigma^{\prime }\left(z\right)=\frac{e^{-z}}{{\left(1+e^{-z}\right)}^{2}}=\delta \left(z\right)\left(1-\delta \left(z\right)\right). \end{array}$$

The sigmoid function curve is smoother if the value of *x* is large or small, which specifies that the derivative *σ*′(*x*) is inclined closely to zero. The model needs to predict two cases in the dichotomy situations. For each of these categories, the prediction probabilities are *p* and 1 − *p*. The expression of cross-entropy loss function at this time is given as

(17) $$\begin{array}{c} L=\frac{1}{N}\sum_{i}L_{i}=\frac{1}{N}\sum_{i}-\left[y_{i}.\;\log\left(p_{i}\right)+\left(1-y_{i}\right).\;\log\left(1-p_{i}\right)\right], \end{array}$$

where the label of sample *i* is indicated by *y*_*i*_, negative and positive classes are indicated by 0 and 1, and *p*_*i*_ represents the likelihood that the sample *i* is anticipated to be positive.

#### 2.2.4. Incorporation of the Multihead Attention Mechanism Scheme

**Figure 9:**
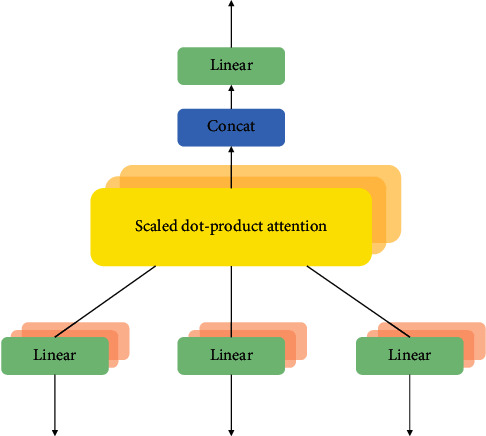
Multihead attention scheme.

A famous brain signal processing procedure similar to vision of humans is the visual attention mechanism. In order to procure the specific area that needs to be carefully pivoted on, the global image is scanned quickly by the human vision and is termed as focus of attention. To get more detailed information, attention resources are fully set to this area so that the necessary attention is paid, and the useless information is avoided completely. Therefore, from a huge amount of information, the information with high values can be easily screened out with very limited attention resources. The efficiency and accuracy of visual information processing are improved to a great extent by means of using human visual attention mechanism. To different fields of deep learning, attention mechanism has been applied, such as image processing tasks, NLP tasks, and speech recognition tasks. Therefore, to understand the development of deep learning methodology, the working of attention mechanism is quite important. When similar sentences appear, then the model will be prompted by the attention mechanism to focus more on the words, so that the learning capability along with its generalization ability of the model is enhanced. A very special case of the general attention mechanism is the self-attention mechanism. The attention-related query matrix is represented by *Q*, the key matrix is represented by *K*, and the value matrix is represented by *V*. The condition of *Q*=*K*=*V* is satisfied in the self-attention mechanism. The distance between the words is completely ignored, and the dependency relationship is calculated directly. The internal structure of a sentence can be learnt well, and a good attention can be paid to the interdependence between the internal words. To enhance the learning model ability and increase the neural network interpretability, the RNN is combined with the CNN model.  Figure 9 explains the basic structure of multihead attention. A variation of the general attention is nothing but the scaled dot product attention at the central position. The computation of the scaled dot product attention for given matrices *Q* ∈ *ℜ*^*n∗d*^, *K* ∈ *ℜ*^*n*×*d*^, and *V* ∈ *ℜ*^*n∗d*^ is given as follows:

(18) $$\begin{array}{c} \text{Attention}\left(Q,K,V\right)=\text{softmax}\left(\frac{QK^{T}}{\sqrt{d}}\right)V, \end{array}$$

where the total number of hidden units in the neural network model is expressed as *d*.

The self-attention mechanism is adopted by the multihead attention implying that *Q*=*K*=*V* as projected in Figure 9. Therefore, to apprehend the dependencies within a full series pattern, the calculation of the current position information along with the other position's information is done because of this mechanism. For instance, if the input is considered as a sentence, then every word in it should be managed with attention calculation. On the inputs *Q*, *K*, and *V*, a linear transformation is performed by the multihead attention. The scaled dot product attention computation is performed multiple times as it is a multihead attention mechanism [40]. For every head calculation, the linear projections of *Q*, *K* and *V* are quite divergent from each other. The number of calculations is actually meant by the number of heads. If the *i*^*th*^ head is considered as an example, then it is represented as follows:

(19) $$\begin{array}{c} Q^{\prime }=Q*W_{i}^{Q},\\ K^{\prime }=K*W_{i}^{K},\\ V^{\prime }=V*W_{i}^{V}. \end{array}$$

The output of the BiGRU layer is received by this layer, and so it is represented as

(20) $$\begin{array}{c} Q=K=V=y_{t}. \end{array}$$

The ultimate result of this head is represented as

(21) $$\begin{array}{c} M_{i}=\text{softmax}\left(\frac{Q^{\prime }{K^{\prime }}^{T}}{\sqrt{d}}\right)V^{\prime }. \end{array}$$

## 3. Results and Discussion

In this section, the evaluation indices and datasets utilized along with the respective analysis of the two proposed deep learning models is analyzed comprehensively.

### 3.1. Evaluation Index

The evaluation indices considered in this work are accuracy, precision, recall, and F score. Their respective formulae are as follows:

(22) $$\begin{array}{c} \text{accuracy}=\frac{T_{P}+T_{N}}{T_{P}+F_{N}+T_{N}+F_{P}},\\ \text{recall}=\frac{T_{P}}{T_{P}+F_{N}},\\ \text{precision}=\frac{T_{P}}{T_{P}+F_{p}},\\ F_{1}=\frac{2*\text{precision}*\text{recall}}{\text{precision}+\text{recall}}, \end{array}$$

where TP, TN, FP, and FN represent true positive, true negative, false positive, and false negative, respectively.

### 3.2. Datasets Utilized

In order to conduct the performance evaluation of the proposed approach for medical text classification, the experiments were tested on two important benchmarking medical literature datasets, such as the Hallmarks dataset and AIM dataset. These datasets are available in [17] and is nothing but a group of biomedical publication abstracts, which are annotated for the hallmark of cancer. In about 1852 biomedical publication abstracts, three hallmarks of cancer are contained in this dataset, such as activating invasion and metastasis, deregulating cellular energetics, and tumor promoting inflammation. AIM dataset, known as activating invasion, and metastasis database has two sets of categories, such as positive and negative. All the in-depth details of the dataset can be obtained in [17]. The details of the datasets are tabulated in Table 1.

### 3.3. Analysis with Proposed Model 1: Quad Channel Hybrid LSTM

For the proposed quad channel hybrid LSTM, the experiments were analyzed with various parameters in order to get the consolidated best results. The batch size was set as 512, and the filter size was chosen in the ranges between [1, 3, 5, 7, 9]. The feature map number was assigned as 200, and the activation function was experimented with various combinations, such as ReLU, sigmoid, SoftPlus, and hard sigmoid. The values of the LSTM output were set as 128, respectively. The learning rate analyzed in this experiment is tested with various values, such as 0.1, 0.01, 0.001, and 0.0001, in order to assess the performance, and the dropout rate was analyzed with 0.2, 0.3, 0.4, and 0.5, to check out for the best results. The loss considered is binary cross entropy and the optimizer utilized is again experimented with Adam, SGD, Nadam, and AdaGrad, to provide a comprehensive analysis.

The experiment was tried for various convolution kernels, and the results are reported in Table 2. The best results are obtained when the kernel filter size is set as [1, 3, 5] as an accuracy of 72.18, precision of 70.52, recall value of 69.69. and *F*1-score of 70.10 are obtained for the Hallmarks dataset, and an accuracy of 94.12, precision of 85.71, recall value of 83.93, and *F*1-score of 84.81 are obtained for the AIM dataset. If the kernel filter size is further increased, then it leads to degradation in the performance computation measures.

The convergence of the objective function to the local minimum is determined by the learning rate, and it serves as a significant hyper parameter in almost all the deep learning applications. In order to make sure that the convergence of the objective function is successfully implemented to the local minimum in a specific interval of time, choosing the learning rate should be done wisely. The convergence would be very slow or moderate if the learning rate is quite small. A cost function oscillation will occur if the learning rate is too high. The experiment was tried for different learning rates, and the results are reported in Table 3. The best results are obtained when the learning rate is set as 0.01 as an accuracy of 73.18, precision of 71.95, recall value of 72.67, and *F*1-score of 72.30 are obtained for the Hallmarks dataset, and an accuracy of 95.72, precision of 86.77, recall value of 83.98, and *F*1-score of 85.35 are obtained for the AIM dataset. The experiment was started with the learning rate of 0.1, but it did not provide satisfactory results. However, when leaning rate was set as 0.01, the best result was obtained and if the learning rate is further decreased, then there is a degradation in the performance metrics measures.

The experiment was tried for different dropout rates, and the results are reported in Table 4. The best results are obtained when the dropout rate was gradually increased from 0.2 to 0.5. When the dropout rate was set as 0.5, the best results are obtained, and an accuracy of 72.93, precision of 70.95, recall value of 69.67, and *F*1-score of 70.30 are obtained for the Hallmarks dataset, and an accuracy of 94.73, precision of 87.81, recall value of 88.98, and *F*1-score of 88.39 are obtained for the AIM dataset.

Table 5 shows the analysis of results with different optimizers for the proposed quad channel hybrid model. The best results are obtained when Adam optimizer is used instead of SGD, Nadam, and AdaGrad as a high accuracy of 72.98, precision of 69.65, recall value of 71.61, and *F*1-score of 70.61 are obtained for the Hallmarks dataset, and an accuracy of 95.12, precision of 87.17, recall value of 85.99, and *F*1-score of 86.57 are obtained for the AIM dataset.

Table 6 shows the analysis of results with different activation functions for the proposed quad channel hybrid model. The best results are obtained when ReLU activation function is used instead of Sigmoid, SoftPlus, and hard sigmoid as a high accuracy of 71.92, precision of 70.92, recall value of 68.62, and *F*1-score of 69.75 are obtained for the Hallmarks dataset, and an accuracy of 92.17, precision of 88.83, recall value of 86.91, and *F*1-score of 87.85 are obtained for the AIM dataset.

Table 7 shows the consolidated analysis of the proposed quad channel hybrid model with the best combinations of values. The best results are obtained when the convolution filter size is [1, 3, 5], learning rate is 0.01, dropout rate is 0.5, optimization function used in Adam along with ReLU activation function is used instead of Sigmoid, SoftPlus, and hard sigmoid, and the results are interpreted. The proposed quad channel LSTM model produces an accuracy of 75.98, precision of 71.95, recall value of 70.67, and *F*1-score of 71.30, which are obtained for the Hallmarks dataset, and an accuracy of 96.72, precision of 88.87, recall value of 86.98, and *F*1-score of 87.91 are obtained for the AIM dataset.

### 3.4. Analysis with Proposed Model 2: Hybrid BiGRU Model with Multihead Attention

The analysis with the proposed second model deals with analysis of various filter sizes of CNN, analysis with different layers of BiGRU, analysis with different learning rates, analysis with different dropout rates, analysis with different optimizers, and analysis with different activation functions. To test the model, various convolution kernel filter sizes were effectively utilized, and the results are tabulated in Table 8. The efficiency of the model training will be greatly affected by the different kernel sizes, and so, the accuracy of the experimental results can vary. The best results are obtained when the kernel filter size is set as [1, 3, 5] as an accuracy of 73.88, precision of 69.54, recall value of 68.67, and *F*1-score of 69.10 are obtained for the Hallmarks dataset, and an accuracy of 95.29, precision of 89.88, recall value of 84.13, and *F*1-score of 86.90 are obtained for the AIM dataset. If the kernel filter size is further increased, then it leads to degradation in the performance computation measures.

For the developed hybrid BiGRU model, the analysis is done with a single layer and multiple layers too, and the analysis of these results is tabulated in Table 9. The best results are obtained when the number of layers is set as two, as an accuracy of 72.11, precision of 71.87, recall value of 72.75, and *F*1-score of 72.30 are obtained for the Hallmarks dataset, and an accuracy of 94.01, precision of 88.95, recall value of 84.91, and *F*1 score of 86.88 are obtained for the AIM dataset. If the layer size is slightly increased, then it leads to a degradation in the performance computation measures.

Multiple learning rates are assessed in this experiment for this architecture also, so that an optimal learning rate is found out, and the results are tabulated in Table 10. The best results are obtained when the learning rate is set as 0.01, as an accuracy of 74.18, precision of 73.58, recall value of 72.93, and *F*1-score of 73.25 are obtained for the Hallmarks dataset, and an accuracy of 95.12, precision of 87.87, recall value of 87.27, and *F*1-score of 87.56 are obtained for the AIM dataset. The experiment was started with the learning rate of 0.1 but it did not provide satisfactory results. However, when leaning rate was set as 0.01, the best result was obtained. If the learning rate is further decreased, then there is degradation in the performance metrics measures, similar to the proposed first model.

The experiment was tried for different dropout rates, and the results are reported in Table 11. The best results are obtained when the dropout rate was gradually increased from 0.2 to 0.5. When the dropout rate was set as 0.4, the best results are obtained, and an accuracy of 72.82, precision of 71.56, recall value of 70.92, and *F*1-score of 71.23 are obtained for the Hallmarks dataset, and an accuracy of 94.22, precision of 89.87, recall value of 88.21, and *F*1-score of 86.12 are obtained for the AIM dataset.

Table 12 shows the analysis of results with different optimizers for the proposed hybrid BiGRU model. The best results are obtained when Adam optimizer is used instead of SGD, Nadam, and AdaGrad, as a high accuracy of 70.52, precision of 74.78, recall value of 73.16, and *F*1-score of 73.96 are obtained for the Hallmarks dataset, and an accuracy of 93.98, precision of 88.63, recall value of 89.08, and *F*1-score of 88.85 are obtained for the AIM dataset.

Table 13 shows the analysis of results with different activation functions for the proposed quad channel hybrid model. The best results are obtained when sigmoid activation function is used instead of ReLU, SoftPlus, and hard sigmoid, as a high accuracy of 74.69, precision of 72.29, recall value of 71.91, and *F*1-score of 72.09 are obtained for the Hallmarks dataset, and an accuracy of 94.29, precision of 89.74, recall value of 88.56, and *F*1-score of 89.14 are obtained for the AIM dataset.

Table 14 shows the consolidated analysis of the proposed hybrid BiGRU model with the best combinations of values. The best results are obtained when the convolution filter size is [1, 3, 5], learning rate is 0.01, dropout rate is 0.4, optimization function used is Adam along with sigmoid activation function instead of ReLU, SoftPlus, and hard sigmoid, and the results are interpreted. The proposed hybrid BiGRU model produces an accuracy of 74.71, precision of 70.82, recall value of 68.99, and *F*1-score of 69.89, which are obtained for the Hallmarks dataset, and an accuracy of 95.76, precision of 88.38, recall value of 84.15, and *F*1-score of 86.21 are obtained for the AIM dataset.

### 3.5. Baseline Methods and Overall Comparison with Other Methods

For text classification, the following baseline methods are used for comparison, such as CNN, LSTM, BiLSTM, CNN-LSTM, CNN-BiLSTM, logistic regression, naïve Bayesian classifier (NBC), SVM, and BiGRU.  Table 15 reports the compared classification accuracy of the two proposed architectures against other machine and deep learning models on the two datasets. Actually, just one or two works published in high quality peer-reviewed journals are available online for comparison of the proposed deep learning models with the results of the other deep learning models on the same dataset. Therefore, in this work, the results have been computed and then compared by analyzing the proposed deep learning model results with the standard and conventional deep learning techniques.

The developed two models have obtained very good results and crossed the performance of the state of art literature compared with some deep learning models. In machine learning and deep learning, it has to be observed that the final classification accuracies may range from a plus or minus two to three percent, but the working methodology and interpretation of the result are more important than trying to prove or obtain slightly higher classification accuracy than the other methods. Therefore, with this understanding the proposed quad channel hybrid LSTM model produced a high classification accuracy of 75.98% for the Hallmark dataset, and the same model produced a classification accuracy of 96.72% for the AIM dataset. The high performance is due to the development of four channels, so that the inherent features can be learnt and observed well through those channels, thereby enhancing the characteristic diversity of the input. Similarly, the hybrid BiGRU with multihead attention model produced a high classification accuracy of 74.71% for the Hallmark dataset, and the same model produced a classification accuracy of 95.76% for the AIM dataset. This is due to the effective capturing of the features by the hybrid model along with the careful selection of appropriate hyperparameters.

## 4. Conclusion and Future Work

By means of extracting the structured information, such as specification of the diseases and the pathological conditions associated with it, the information embedded in the clinical text is unlocked by using automated clinical text classification. By means of using symbolic techniques/statistical techniques, the tackling of the medical text classification is done. Handcrafted expert rules are usually needed every time with symbolic techniques, and they are quite expensive and cumbersome to develop. Statistical techniques, like machine learning, seem to be quite effective for the medical text classification tasks. However, it still requires extensive human efforts in order to label a large set of training data. In this paper, two deep learning models have been developed, and it has been successfully validated on two datasets too. When the proposed quad channel hybrid LSTM is implemented to Hallmarks dataset, a classification accuracy of 75.98% is obtained, and when it is implemented to AIM dataset, a classification accuracy of 96.72% is obtained. When the proposed hybrid BiGRU model is implemented to Hallmarks dataset, a classification accuracy of 74.71% is obtained, and when it is implemented to AIM dataset, a classification accuracy of 95.76% is obtained. Future works aim to develop more effective hybrid deep learning models for the efficient classification of medical texts. Future works also aim to explore content-based features and a variety of other domain specific features and plans to amalgamate it with very efficient hybrid deep learning techniques to get a good classification accuracy.

## Figures and Tables

**Table 1:** Dataset details.

Datasets	Classes	Sentence length	Vocabulary size	Dataset size	Training set	Validation set	Test set
Hallmarks	3	833	29141	8472	5931	1694	847
AIM	2	833	29141	2646	1853	529	264

**Table 2:** Analysis with different convolution kernel conditions for the proposed quad channel hybrid model.

Kernel filter sizes	Dataset	Accuracy (%)	Precision (%)	Recall (%)	*F*1-score (%)
[1, 3]	Hallmarks	62.58	55.76	54.82	55.28
	AIM	81.44	75.36	79.21	77.23

[1, 3, 5]	Hallmarks	72.18	70.52	69.69	70.10
	AIM	94.12	85.71	83.93	84.81

[1, 3, 5, 7]	Hallmarks	71.49	62.98	64.98	63.96
	AIM	79.90	73.98	72.81	73.39

[1, 3, 5, 7, 9]	Hallmarks	64.71	59.37	55.91	57.58
	AIM	57.36	52.90	54.27	53.57

**Table 3:** Analysis of results with different learning rates for the proposed quad channel hybrid model.

Learning rate	Dataset	Accuracy (%)	Precision (%)	Recall (%)	*F*1-score (%)
0.1	Hallmarks	59.85	61.76	60.78	61.26
	AIM	75.47	78.31	79.16	78.73

0.01	Hallmarks	73.18	71.95	72.67	72.30
	AIM	95.72	86.77	83.98	85.35

0.001	Hallmarks	72.19	70.99	72.98	71.97
	AIM	84.65	79.99	79.81	79.89

0.0001	Hallmarks	71.73	74.31	73.81	74.05
	AIM	82.36	80.91	79.73	80.31

**Table 4:** Analysis of results with different dropout rates for the proposed quad channel hybrid model.

Dropout rate	Dataset	Accuracy (%)	Precision (%)	Recall (%)	*F*1-score (%)
0.2	Hallmarks	69.75	61.65	62.89	62.26
	AIM	90.17	87.38	85.12	86.23

0.3	Hallmarks	71.76	63.63	61.79	62.69
	AIM	56.23	51.80	52.67	52.23

0.4	Hallmarks	71.19	65.99	63.18	64.55
	AIM	79.71	73.99	72.91	73.44

0.5	Hallmarks	72.93	70.95	69.67	70.30
	AIM	94.73	87.81	88.98	88.39

**Table 5:** Analysis of results with different optimizers for the proposed quad channel hybrid model.

Optimizer	Dataset	Accuracy (%)	Precision (%)	Recall (%)	*F*1-score (%)
Adam	Hallmarks	72.98	69.65	71.61	70.61
	AIM	95.12	87.17	85.99	86.57

SGD	Hallmarks	71.72	63.58	61.84	62.69
	AIM	82.35	78.98	77.21	78.08

Nadam	Hallmarks	71.43	65.98	63.28	64.60
	AIM	86.76	72.98	72.31	72.64

AdaGrad	Hallmarks	70.61	61.56	60.81	61.18
	AIM	94.41	85.13	85.26	85.19

**Table 6:** Analysis of results with different activation functions for the proposed quad channel hybrid model.

Optimizer	Dataset (%)	Accuracy (%)	Precision (%)	Recall (%)	*F*1-score (%)
ReLU	Hallmarks	71.92	70.92	68.62	69.75
	AIM	92.17	88.83	86.91	87.85

Sigmoid	Hallmarks	69.71	64.72	60.81	62.70
	AIM	55.31	50.90	52.21	51.54

SoftPlus	Hallmarks	70.49	64.97	66.91	65.92
	AIM	77.73	74.91	72.82	73.85

Hard sigmoid	Hallmarks	64.35	61.75	60.81	61.27
	AIM	89.48	86.22	85.22	85.71

**Table 7:** Consolidated results for the best combination values of the proposed quad channel hybrid model.

Model	Dataset	Accuracy (%)	Precision (%)	Recall (%)	*F*1-score (%)
Quad channel LSTM	Hallmarks	75.98	71.95	70.67	71.30
	AIM	96.72	88.87	86.98	87.91

**Table 8:** Analysis with different convolution kernel conditions for the proposed hybrid BiGRU model.

Kernel filter sizes	Dataset	Accuracy (%)	Precision (%)	Recall (%)	*F*1-score (%)
[1, 3]	Hallmarks	71.85	61.79	60.70	61.24
	AIM	93.27	89.36	85.29	87.27

[1, 3, 5]	Hallmarks	73.88	69.54	68.67	69.10
	AIM	95.29	89.88	84.13	86.90

[1, 3, 5, 7]	Hallmarks	72.52	69.72	65.99	67.80
	AIM	82.79	76.91	73.78	75.31

[1, 3, 5, 7, 9]	Hallmarks	70.48	67.72	63.85	65.72
	AIM	58.39	51.84	57.18	54.37

**Table 9:** Analysis of results with different BiGRU layers.

Layers	Dataset	Accuracy (%)	Precision (%)	Recall (%)	*F*1-score (%)
1	Hallmarks	67.81	59.16	57.19	58.15
	AIM	91.11	83.35	83.26	83.30

2	Hallmarks	72.11	71.87	72.75	72.30
	AIM	94.01	88.95	84.91	86.88

3	Hallmarks	71.91	69.27	65.91	67.54
	AIM	81.16	76.18	75.37	75.77

4	Hallmarks	72.73	66.17	64.98	65.56
	AIM	69.69	54.99	57.21	56.07

**Table 10:** Analysis of results with different learning rates.

Learning rate	Dataset	Accuracy (%)	Precision (%)	Recall (%)	*F*1-score (%)
0.1	Hallmarks	63.85	56.16	55.81	55.98
	AIM	88.17	82.36	81.28	81.81

0.01	Hallmarks	74.18	73.58	72.93	73.25
	AIM	95.12	87.87	87.27	87.56

0.001	Hallmarks	74.41	69.91	69.98	69.94
	AIM	82.64	77.09	76.91	76.99

0.0001	Hallmarks	70.71	64.71	65.91	65.30
	AIM	61.36	55.16	57.73	56.41

**Table 11:** Analysis of results with different dropout rates for the proposed hybrid BiGRU model.

Dropout rate	Dataset	Accuracy (%)	Precision (%)	Recall (%)	*F*1-score (%)
0.2	Hallmarks	61.88	57.19	58.84	58.00
	AIM	89.72	83.58	82.97	83.27

0.3	Hallmarks	71.51	66.73	64.70	65.69
	AIM	62.39	57.18	55.23	56.18

0.4	Hallmarks	72.82	71.56	70.92	71.23
	AIM	94.22	89.87	88.21	86.12

0.5	Hallmarks	72.45	68.97	67.88	68.42
	AIM	81.61	75.98	74.92	75.44

**Table 12:** Analysis of results with different optimizers for the proposed hybrid BiGRU model.

Optimizer	Dataset	Accuracy (%)	Precision (%)	Recall (%)	*F*1-score (%)
Adam	Hallmarks	70.52	74.78	73.16	73.96
	AIM	93.98	88.63	89.08	88.85

SGD	Hallmarks	69.72	65.23	61.83	63.48
	AIM	69.31	66.57	68.27	67.40

Nadam	Hallmarks	70.13	68.84	68.64	68.73
	AIM	80.62	75.96	72.56	74.22

AdaGrad	Hallmarks	65.15	62.46	55.81	58.94
	AIM	86.15	81.59	81.28	81.43

**Table 13:** Analysis of results with different activation functions for the proposed hybrid BiGRU model.

Optimizer	Dataset	Accuracy (%)	Precision (%)	Recall (%)	*F*1-score (%)
ReLU	Hallmarks	71.64	67.39	66.69	67.03
	AIM	88.39	82.20	83.61	82.89

Sigmoid	Hallmarks	74.69	72.29	71.91	72.09
	AIM	94.29	89.74	88.56	89.14

SoftPlus	Hallmarks	73.12	71.03	71.82	71.42
	AIM	83.39	81.91	82.84	82.37

Hard sigmoid	Hallmarks	61.22	58.92	57.41	58.15
	AIM	85.87	83.19	81.49	82.33

**Table 14:** Comparison of results for the best combination values of the proposed BiGRU hybrid model.

Model	Dataset	Accuracy (%)	Precision (%)	Recall (%)	*F*1-score (%)
Hybrid BiGRU model	Hallmarks	74.71	70.82	68.99	69.89
	AIM	95.76	88.38	84.15	86.21

**Table 15:** Performance comparison of accuracy (%) of the proposed deep learning models with other deep learning models on the same dataset.

Methods	Hall mark dataset	AIM dataset
CNN	68.55	82.17
LSTM	70.76	83.16
BiLSTM	72.58	87.77
CNN-LSTM	71.81	91.98
CNN-BiLSTM	73.99	93.06
Logistic regression	61.91	72.92
NBC	65.35	73.84
SVM	66.99	84.55
BiGRU	69.34	89.98
Proposed method 1: quad channel hybrid LSTM model	75.98	96.72
Proposed method 2: hybrid BiGRU with multihead attention model	74.71	95.76

## Data Availability

All the programming codes will be made available to the researchers upon request to the corresponding author.
